# Clustered Regularly Interspaced Short Palindromic Repeats/Cas9 Gene Editing Technique in Xenotransplantation

**DOI:** 10.3389/fimmu.2018.01711

**Published:** 2018-09-05

**Authors:** Meisam Naeimi Kararoudi, Seyyed S. Hejazi, Ezgi Elmas, Mats Hellström, Maryam Naeimi Kararoudi, Arvind M. Padma, Dean Lee, Hamid Dolatshad

**Affiliations:** ^1^The Childhood Cancer Center at Nationwide Children’s Hospital, Columbus, OH, United States; ^2^Department of Basic Science of Veterinary Medicine, Tabriz Branch, Islamic Azad University, Tabriz, Iran; ^3^Laboratory for Transplantation and Regenerative Medicine, Department of Obstetrics and Gynecology, Sahlgrenska Academy, University of Gothenburg, Gothenburg, Sweden; ^4^Eye Research Center, Farabi Eye Hospital, Tehran University of Medical Science, Tehran, Iran; ^5^Bloodwise Molecular Haematology Unit, Nuffield Division of Clinical Laboratory Sciences, Radcliffe Department of Medicine, University of Oxford, Oxford, United Kingdom

**Keywords:** clustered regularly interspaced short palindromic repeats/Cas9, xenotransplantation, zinc finger nucleases–transcription activator-like effector nuclease–clustered regularly interspaced short palindromic repeats/Cas, transplantation immunology, gene editing

## Abstract

Genetically modified pigs have been considered favorable resources in xenotransplantation. Microinjection of randomly integrating transgenes into zygotes, somatic cell nuclear transfer, homologous recombination, zinc finger nucleases, transcription activator-like effector nucleases, and most recently, clustered regularly interspaced short palindromic repeats-cas9 (CRISPR/Cas9) are the techniques that have been used to generate these animals. Here, we provide an overview of the CRISPR approaches that have been used to modify genes which are vital in improving xenograft survival rate, including cytidine monophosphate-*N*-acetylneuraminic acid hydroxylase, B1,4N-acetylgalactosaminyltransferase, isoglobotrihexosylceramide synthase, class I MHC, von Willebrand factor, C3, and porcine endogenous retroviruses. In addition, we will mention the importance of potential candidate genes which could be targeted using CRISPR/Cas9.

## Introduction

Xenotransplantation is a potential solution for the urgent and steadily increasing worldwide persisting donor organ shortage (1). Since the 1900s, several efforts have been made in xenotransplantation using animal organs derived from pigs, goats, lambs, or monkeys, but none of them were successful (2). Several studies have shown that pigs are the best choice of source for providing the limitless organs on demand for human (3). The four most significant and profound barriers to organ xenotransplantation are the immunologic responses to the porcine grafted organs, namely, hyperacute rejection (HAR), acute humoral xenograft rejection (AHXR), immune cell-mediated rejection, and chronic rejection (4). To overcome xenograft rejection and the barriers mentioned above, several investigations using pig-to-baboon models has been performed (5). Heterotopic and intrathoracic heterotopic cardiac xenograft in a combination with potent immunosuppression therapy have survived beyond 900 days (5, 6). Undoubtedly, genetic engineering has been the most important factor in these achievements by producing genetically modified pigs which are more compatible and acceptable for the human immune system. The most recent genetic technique, clustered regularly interspaced short palindromic repeats-cas9 or CRISPR/Cas9 has been a milestone in gene editing so far, particularly in xenotransplantation (7). This review provides an overview of achievements and perspectives of the newest approach “CRISPR/Cas9” that is used to generate organ donor pigs for xenotransplantation.

## CRISPR/Cas9

**Figure 1:**
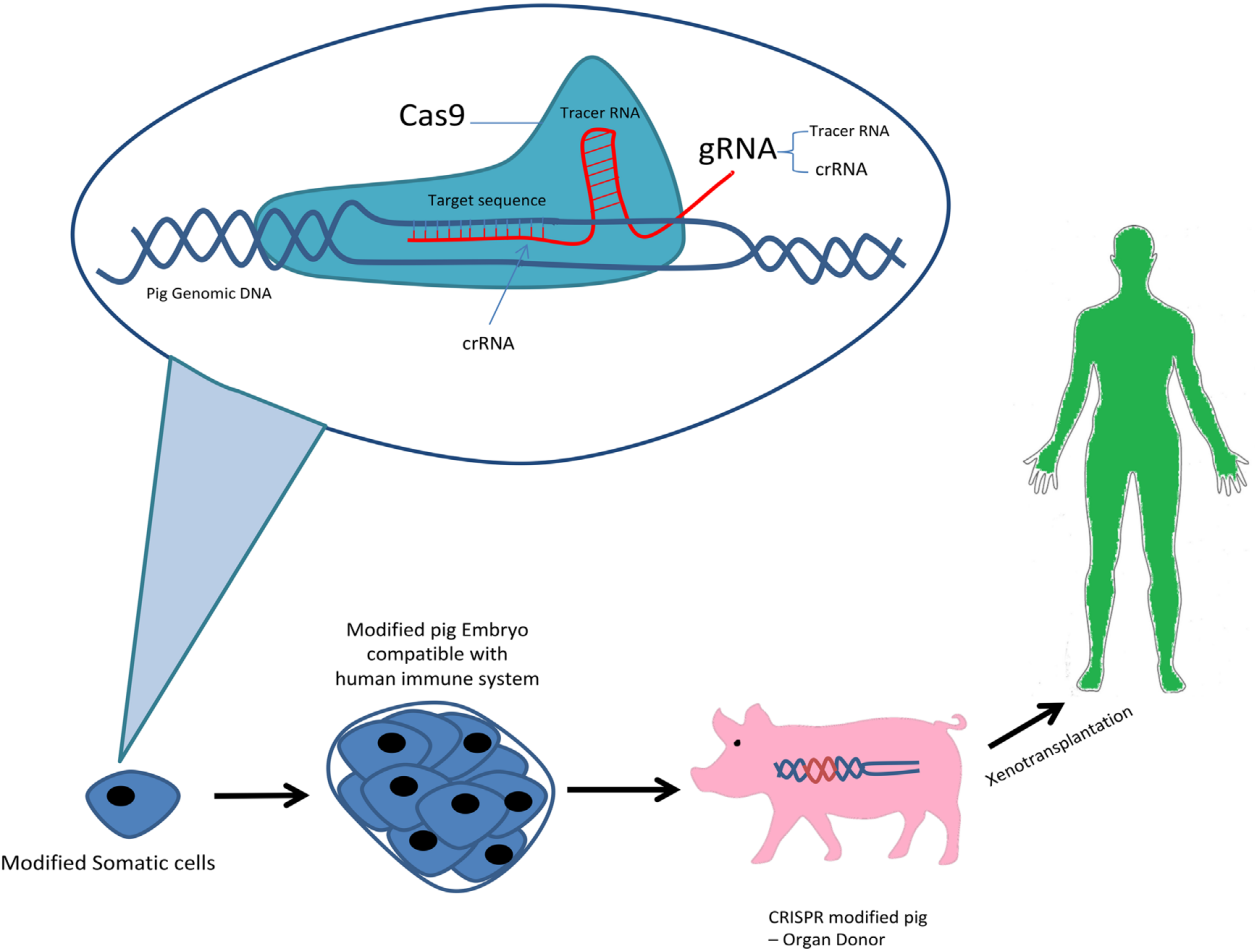
CRISPR system in xenotransplantation.

Clustered regularly interspaced short palindromic repeats/Cas9 genome editing system was first discovered as an RNA-guided defense mechanism for bacteria against foreign genetic elements by viruses or phages in order to respond and eliminate their invading genetic elements. There exists three types of CRISPR mechanisms and the type II CRISPR system has been studied the most. The simplicity of the type II CRISPR nuclease system consisting of three components Cas9 protein, the CRISPR RNA (crRNA) and a trans-activating crRNA (tracrRNA) has allowed it to be used as the most favorable genome editing system to date. The type II requires a 20-nucleotide guide sequence as part of the crRNA and tracrRNA partially complementary to the crRNA, and a Cas9 endonuclease protein to cleave the genomic DNA. Recent advances has allowed synthesis of single-guide RNA (sgRNA) consisting of a fusion of crRNA and tracrRNA *in vitro*. In the Cas9 nuclease protein in type II CRISPR/Cas system can cleave target DNA at specific sites producing a double-stranded DNA break. Following the DNA break, two repair mechanisms exist, non-homologous end joining which is error prone and can lead to mutations by insertion/deletion (indel) or homology-directed repair which is an alternative DNA repair mechanism, in the presence of a repair template, precise and defined modifications are generated at the DNA target sites. Recent improvement in the technology and research has lead to novel ideas such as, CRISPR/Cpf1 genome editing system of the bacterium *Francisella novicida*, CRIPSR Ribonucleoprotein (RNP) and CRISPRa/i (activation/interference) (8). So far, the CRIPSR/Cas9 system has been the only one used in pig genome editing. In 2014, the first use CRISPR/Cas 9 to generate pig’s knockout cells, demonstrated its potential in complex genome engineering (9) (Figure 1).

## CRISPR/Cas9 Modified Genes

Modifying the genes which cause the immunologic responses to the porcine grafted organs, namely, HAR, AHXR, immune cell-mediated rejection, and chronic rejection have enhanced survival rate of organ xenotransplantation. Here, after introducing the importance of the CRISPR modified genes viz. cytidine monophosphate-*N*-acetylneuraminic acid hydroxylase (CMAH), B1,4N-acetylgalactosaminyltransferase (B4GALNT2), isoglobotrihexosylceramide synthase (iGb3s), class I MHC, von Willebrand factor (vWF), C3, and porcine endogenous retroviruses (PERVs) in xenotransplantation, we will describe the different approaches which have been used for applying CRISPR/Cas9 to target these genes (Table 1).

**Table 1:** Clustered regularly interspaced short palindromic repeats/Cas9 modified genes and their benefits for xenotransplantation.

Name of the modified gene	Benefits for xenotransplantation	Reference
Alpha-Gal expression (GGTA1)	Reduction of formation of the membrane attack complex (MAC)	(9)
Cytidine monophosphate-*N*-acetylneuraminic acid hydroxylase (CMAH)	Reduction of formation of the MAC	(11)
B1,4N-acetylgalactosaminyltransferase (B4GALNT2)	Reduction of human antibody-mediated cytotoxicity	(12, 13)
Isoglobotrihexosylceramide synthase (iGb3s)	Understanding its role in Gal-related xenograft rejection	(24)
Class I MHC	Improving the ability of study class I MHC function in pigs	(25)
von Willebrand factor (vWF)	Reduction of formation of activated platelets	(28)
Complement component (C3)	Reduction of formation of complement system	(29, 34)
Porcine endogenous retroviruses (PERVs)	Reduction of human cell infection by releasing PERVs	(32)

## Alpha-Gal Expression (GGTA1)

The porcine *GGTA1* encodes a 371-amino acid protein that synthesizes a sugar epitope present on the surface of all porcine cells but not in old world monkeys such as baboons or humans. This protein belongs to glycosyltransferase 6 family and transfers galactose from UDP-galactose to an acceptor molecule. Natural production of antibodies against alpha-1,3-Gal in humans and baboons leads to the formation of the membrane attack complex that causes HAR by interstitial hemorrhage and edema through activation of several complement reactions. These responses lead to graft vasculature destruction, and graft failure happens within seconds and hours after transplantation (4, 10). Alpha-1,3-galactosyl transferase is encoded by 6 exons (4–9), exon 4 contains the endogenous ATG translational initiation codon and exon 9 that codes the catalytic domain. The last exon covers most of the protein (amino acids 141 to 371), including the active domain with a-1,3-galactosyl transferase activity.

The first try to destroy the function of porcine *GGTA1* using the CRISPR/Cas9 system was done by Sato et al. in 2014 (9). They obtained biallelic KO cells for *GGTA1* by the combined use of the CRISPR/Cas9 system with targeted toxin-based selection [IB4 conjugated with saporin (IB4SAP)]. In their study, a pair of gRNA oligos were designed and cloned into an hCas9 expression vector carrying a codon-optimized Cas9 gene for targeting the exon 4 of *GGTA1* in porcine embryonic fibroblast (PEFs). PEFs were electroporated in nucleofector solution (for primary fibroblasts) containing the hCas9 expression vector, gRNA expression vector, and pmaxGFP. Approximately 90% of colonies that survived after IB4SAP treatment were α-Gal epitope negative. In another attempt in 2014, Li et al. generated genetically distinct pigs in a single pregnancy using multiplexed sgRNA and carbohydrate selection. They used the magnetic beads to separate the cells and made a model for targeting three genes including *GGTA1, CMAH*, and *iGb3s* (11). Other independent groups used a similar method for knocking out *GGAT1* to test human-anti-pig cytotoxicity (12–14). In another attempt, the exon 8 of *GGAT1* were targeted to produce KO pigs using microinjection of GGTA1-CRISPR/Cas9 using px330 expression vector to transduce PEFs (15).

Interestingly, Su et al. (16) in 2015 improved the efficiency of targeting pig genome. They developed a CRISPR–Cas9 system that was particularly adaptive in porcine PK1 cells. They flanked the SV40 T-antigen NLS (PKKKRKVG, NLS1) and the Dax NLS (KKSRKEKK, NLS2) at the N and C termini, respectively, to the *Streptococcus pyogenes* A20 Cas9 with the humanized codon. An overlapped Flag2 tag (EYKDDDGDYKDDDDK) was added at the end of the N terminus. The CMV enhancer-chicken b-actin promoter was used to derive the Flag2-NLS1-Cas9-NLS2mRNA, and the porcine U6 promoter was used to transcribe the spacer-gRNA chimeric RNA. Four target sites within *GGTA1* gene region, including parts of the last intron and last exon, were picked up in this study.

Nevertheless, knocking out is not the only way to reduce alpha-Gal expression. Sato et al. reported the first successful knock-in of a small sequence at an endogenous target (*GGTA1* locus) in porcine cells *via* homologous recombination (HR) by CRISPR/Cas9 system. Due to the generally low efficacy of CRISPR/Cas9-mediated knock-in, they employed IB4SAP as targeted toxin-based drug-free selection system and they significantly improved facilitating the creation of loss-of-function alleles by combining IBS4SAP. In this study, PEFs were transfected by phCas9 and a pE4 plasmid termed pgRNA which carries the specific guide RNA sequence targeted (spanning ~800 bp) at the exon 4 of *GGTA1*. They successfully obtained several knock-in clones within 3 weeks of initial transfection (17).

## Cytidine Monophosphate-*N*-Acetylneuraminic Acid Hydroxylase

In addition to alpha-Gal, the expression of another carbohydrate xenoantigen N-glycolylneuraminic acid (Neu5Gc) is present in pigs, but not in humans. The *CMAH* gene was inactivated like GGTA1 during evolution because of its protective role against a prevailing malaria strain (18). This gene similar to *GGTA1* gene is widely expressed on the endothelial cells of pigs. This epitope can activate anti-non-Gal antibody in humans as it is responsible for the expression of Neu5Gc, a key non-Gal antigen. Human beings express the acetylated form of the sugar (Neu5Ac) instead. It has been hypothesized that elimination of *CMAH* gene expression in pigs is crucial for increasing survival rate of xeno-organ (19–21). Furthermore, the pigs lacking both *GGTA1* and *CMAH* KO genes reduce the humeral barrier to xenotransplantation in comparison to those lacking *GGTA1* alone (22).

For the first time in 2015, CRISPR technology was applied for knocking out the *CMAH* gene. Li et al. used the same knocking out method for the *CMAH* gene as *GGTA1*. They showed that knocking out the *CMAH* using CRISPR/Cas9 is more promising than zinc finger nucleases (ZFN) and transcription activator-like effector nucleases (TALENs) method (11). In addition, *GGTA1* and *CMAH* knockout pigs were produced by the same method (12, 13). In 2017, *GGTA1/CMAH* double knockout pigs were generated *via* “handmade cloning” using CRISPR/Cas9. The *GGTA1* sgRNA targeted exon 6, and the *CMAH* sgRNA targeted exon 1. The Cas9-coding DNA and sgRNAs were cloned in into the pMD-18T vector to modify the genes in Wuzhishan porcine fetal fibroblasts (PFFs) cells (23).

## B1,4N-Acetylgalactosaminyltransferase

B1,4N-acetylgalactosaminyltransferase is a glycosyltransferase that catalyzes the terminal addition of N-acetylgalactosamine to a sialic acid modified lactosamine to produce GalNAcb4 [Neu5Aca2,3] Gal b1-4GlcNAc, b1-3Gal and the Sda (Sid blood group, also known as CAD or CT) blood group antigen. Most humans produce low levels of antibodies to Sda. Therefore, its deletion would be a promising approach to reduce pig organ rejection. Butler et al. described the first characterization of the effect of silencing the *B4GalNT2* gene on human antibody-mediated cytotoxicity. Genetically modified pigs were created utilizing a CRISPR/Cas9 approach transfer as described by Li et al. in 2015 (12, 13).

## Isoglobotrihexosylceramide Synthase

The importance of iGb3s was due to this hypothesis that it might be a source of α-Gal epitopes in *GGTA1*(−/−) animals. *iGb3s* is another member of the glycosyltransferase family that catalyzes the synthesis of isoglobo-series glycosphingolipids with a α-Gal-terminal disaccharide (iGb3). To examine the impact of silencing the *iGb3s* gene (*A3GalT2*) on pig-to-primate and pig-to-human immune cross-reactivity, creating and comparing *GGTA1*(−/−) pigs to *GGTA1*(−/−) and *A3GalT2*(−/−) double knockout pigs, Butler et al. generated the *GGTA1* and *A3GalT2* knock out pigs using CRISPR/Cas9. They showed that iGb3s is not a contributor to antibody-mediated rejection in pig-to-primate or pig-to-human xenotransplantation (24).

## Class I MHC

Class I MHC or Swine Leukocyte Ags (SLA)-null pigs were created using CRISPR/Cas9 system by Reyes et al. in 2014. Classical class I MHC gene synthesizes polymorphic proteins that have several activities such as CD8+ T lymphocyte activation; additionally, they regulate the activity of other immune effectors such as NK cells. The class I region of swine MHC contains three classical class I genes (SLA-1, -2, and -3), several pseudogenes (SLA-4, -5, and -9), and two class I-like genes (SLA-11 and -12). To improve the ability of study class I MHC function in xenograft rejection, authors used the Cas9 nuclease and gRNAs targeting sequences of exon 4 of the class I gene which consists of 276 bp in the different alleles. Following somatic cell nuclear transfer, they created cloned animals lacking class I MHC protein expression. Although these animals have reduced levels of CD4(−)CD8(+) T cells in peripheral blood, the pigs appeared healthy and were developing normally (25).

## von Willebrand Factor

Pig vWF is a glycoprotein that plays a critical role in the pathogenesis of xenograft failure, especially in pulmonary xenotransplantation, because the lung releases more vWF than the heart or kidneys. This multimeric glycoprotein spontaneously aggregates human platelets in the absence of shear stress due to an aberrant interaction through an aberrant between its O-glycosylated A1 domain and platelet glycoprotein Ib (GPIb) receptors (26). After GPIb–vWF interaction happens, intracellular signaling occurs, and platelets become activated. Circulating activated platelets develop thrombus after being recruited to the place of the endothelial cells injury (27).

These incompatibilities can be resolved by the generation of vWF knockout pigs by zygote injection of CRISPR/Cas9 system. Hai et al. in 2014 generated vWF knockout to improve bleeding efficiency for slaughtering procedures and blood collection that could be used for xenotransplantation purposes as well. They designed sgRNA-targeted exon 5 of the pig vWF gene, which lies in the first trysin-inhibitor-like domain and its mutation could lead to the loss of function of vWF protein. Indels were confirmed by the T7E1 assay. The high birth rate (16/76, 21%) and survival rate (14/16, 88%) indicated that Cas9 mRNA/sgRNA had little toxicity to pig embryonic development (28).

## Complement Component (C3)

C3 encoded by the C3 gene is the central component of the complement system as it has a major role in the adaptive immune response. Several tissues and cells have the capacity to produce C3 such as hepatocytes as a primarily source, macrophages, dendritic cells, etc. The activation of complement system can lead to HAR of xenograft. Therefore, its deletion has always been an aim for researchers to produce C3 deficient pigs to reduce complement system. Recently, Zhang and colleagues generated 19 complement protein C3 deficient pigs by CRISPR/Cas9-mediated gene targeting. They transfected PFF cells by a pX330 plasmid that expresses human codon-optimized Cas9 and sgRNA under the chicken beta-actin hybrid and human U6 promoters, respectively. The sgRNAs targeted the exon 26 of C3 gene. They showed that C3 α-chain was undetectable in KO piglets contrary to that of the WT controls. Besides, no complement activity was detected in the serum of C3 KO piglets (29).

## Porcine Endogenous Retroviruses

Porcine endogenous retroviruses are one of the most important challenges in the field of xenotransplantation. Under stress, pig cells can infect human cells invitroby releasing PERVs, but so far there has never been a report of a primate receiving pig cells or tissue being infected by PERVs. These viruses cannot be eliminated by biosecure breeding. ZFN or TALENs were used for inactivating PERVs, but with limited success (30). Inactivating the PERVs by CRIPSR could prevent the transmission of retroviruses *in vitro*. Recently Yang et al. using CRISPR-Cas9, disrupted 62 PERV sites in the animal’s genome and demonstrated a >1,000-fold reduction in PERV transmission from edited porcine cells (PK15) to human cells. Their study demonstrates that CRISPR–Cas9 genome editing system can inactivate PERVs for clinical application of porcine-to-human xenotransplantation. First, they analyzed the sequences of publicly available PERVs and other endogenous retroviruses in pigs to design Cas9 guide RNAs. For having higher editing efficiency by a Cas9 system, they used a PiggyBac transposon system to deliver a doxycycline-inducible Cas9 and the two gRNAs into the genome of PK15 cells (31). The first report on PERV-inactivated pig production using CRISPR/Cas9 was published in Science magazine in August 2017. Niu et al. used the R library DECIPHER to design specific gRNAs that target all pol catalytic sequences in FFF3 cell line. They synthesized a DNA fragment encoding U6-gRNA1-U6-gRNA2 and incorporated it into a previously constructed PiggyBac-cas9 plasmid. By these results, they demonstrated the successful production of PERV-inactivated animals to address the safety concern in clinical xenotransplantation (32).

Considering the above-mentioned achievements, the challenge is to combine multiple genetic modifications to enable normal animal breeding and to defeat rejection mechanisms. To overcome this problem, as the first try Fischer et al. in 2016 produced multi-modified pigs for xenotransplantation by “combineering,” gene stacking and gene editing. They generated new multi-transgenic pigs carrying genomic versions of human complement regulators CD46, CD55, CD59 plus cDNA cassettes for human A20 and HO1 to provide endothelium protection, with all transgenes at a single locus. Later, by using CRISPR/Cas9, biallelic knockout of *GGTA1* and *CMAH* genes24 were then carried out in this multi-transgenic background. A CRISPR/Cas9 enzyme targeted exon 10 of *CMAH* gene in kidney-derived fibroblasts (PKF) that had the multi-transgenic background. Subsequently, *CMAH* knockout clones were used for nuclear transfer; pregnancy was terminated at day 28, fetal fibroblasts isolated and transfected with a CRISPR/Cas9 genome editing system targeting exon 8 of *GGTA1* gene (33).

## Perspective

Besides the CRISPR modified genes, there are other important genes which have been modified in pigs using other gene editing techniques such as HR, ZFN, and TALENS. Recreating these genetically modified pigs by CRISPR/Cas9, regarding its simplicity and accuracy and other benefits, would be considered as a perspective for the future use of this system in xenotransplantation viz. Human CD59+, CD55+, GLA+, H-transferase+, GnT-III+, CD46+, TRAIL+, DAF and MCP+, Porcine CTLA4-Ig+, Human thrombomodulin+, HLA-E/Human Beta-2-microglobulin+, Human A20+, Endo-B-Galactosidase+, CIITA-DN+, Human Fas Ligand+, Human TNFRI-Fc+, Human heme oxygenase 1+, Human CD39+ and LEA29Y+. Table 2 shows the list of these candidate genes and their benefits for xenotransplantation.

**Table 2:** The potential genes for CRISPR modification.

Name of the modified gene	Benefits for xenotransplantation	Reference
Human CD59+	Reduction of activation of serum complement on the luminal surface of the vascular endothelium	(35)
Human CD55+	Reduction of activation of serum complement on the luminal surface of the vascular endothelium	(36)
Human GLA+	Reduction of interaction of Galα(1,3)Gal with antibodies and complement directed against swine Gal antigen	(37)
Human H-transferase+	Reduction of Galα1,3-Gal expression	(38)
Human CD46+	Reduction of activation of serum complement on the luminal surface of the vascular endothelium	(39)
Human GnT-III+	Reduction of antigenicity to natural human antibodies, especially the Galalpha1-3Galbeta1-4GlcNAc-R	(40)
Human TRAIL+	Controlling post-hyperacute rejection mechanisms mediated by cellular components of the immune system	(41)
Human DAF and MCP+	Supporting the idea of modulating coagulation pathway activation in transgenic pigs	(42)
Porcine CTLA4-Ig+	Reduction of T-cell activity	(43)
Human thrombomodulin+	Elevation in activated protein C production to control xenogenic coagulation	(44)
HLA-E/Human Beta-2-microglobulin+	Protection against xenogeneic human anti-pig natural killer cell cytotoxicity	(45)
Human A20+	Protection against apoptotic and inflammatory stimuli	(46)
Endo-B-Galactosidase+	Reduction of alphaGal expression	(47)
CIITA-DN+	Reduction of human CD4(+) T-cell proliferation reduction of humoral and cellular responses to the pig aortic endothelial cells (pAECs)	(48)
Human Fas Ligand+	Reduction of CD8+ CTL-mediated cytotoxicity (49)	(50)
Human TNFRI-Fc+	Reduction of activation of porcine endothelial cells	(51)
Human heme oxygenase 1+	Increasing the protection of xenografts when exposed to oxidative stresses, especially from ischemia/reperfusion injury, and/or acute rejection mediated by cytokines	(52)
Human CD39+	Protection against myocardial injury and ischemia/reperfusion injury	(53)
LEA29Y+	Normalize blood glucose levels and inhibition of human–anti-pig rejection	(54)

## Conclusion

Production of transgenic pigs has helped substantial progress the field of xenotransplantation and created hope that clinical trials may no longer be a distinct prospect. CRISPR/Cas9 technology would likely accelerate these achievements by its ease and precision. New improvements in CRISPR/Cas9 technology such as Cas9/RNP and CRISPR/Cpf1 can accelerate this field (55–57). The CRISPR approaches that were described in this review might advance and help researchers to design their CRISPR/Cas9 project and lead xenotransplantation from bench closer to bedside (58–59).

## Author Contributions

MNK and HD wrote and designed the manuscript. SSH, EE, MH, MNK, DL and AMP reviewd and edited the manuscript

## Conflict of Interest Statement

The authors declare that the research was conducted in the absence of any commercial or financial relationships that could be construed as a potential conflict of interest.
